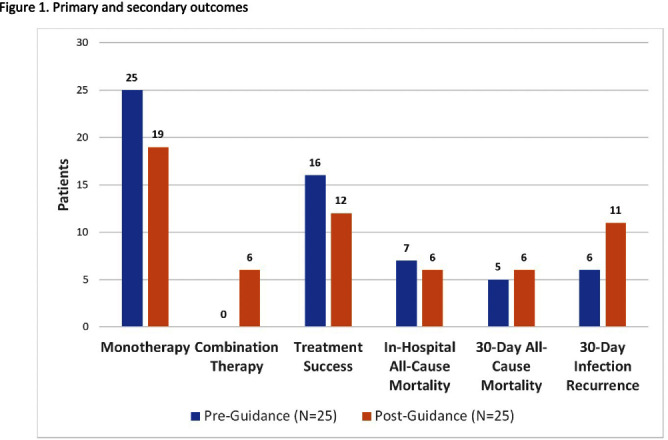# Evaluation of Practice Changes in Therapy for Stenotrophomonas maltophilia

**DOI:** 10.1017/ash.2024.265

**Published:** 2024-09-16

**Authors:** Alex Peterson-Weber, Kendall Donohoe, Christopher McCoy

**Affiliations:** Beth Israel Deaconess Medical Center

## Abstract

**Background:** Stenotrophomonas maltophilia (SM) is a non-fermenting, Gram-negative bacillus. Its intrinsic resistance to many beta-lactams makes for challenging treatment decisions. A preprint of the latest Infectious Diseases Society of America (IDSA) guidance on managing SM infections was published in December 2022 providing a recommendation for combination therapy including trimethoprim/sulfamethoxazole (TMP/SMX) and a second agent. An evaluation of the impact on SM treatment practices following this guidance was conducted at our institution. **Methods:** A list of 130 patients with non-urine SM cultures from December 2021-August 2023 was generated using a pharmacovigilance platform. Patients were excluded if on comfort measures or discharged to hospice prior to therapy completion, no directed antibiotics were given, or any history of prior SM infection. Twenty-five patients were randomly selected from the pre- and post-guidance periods (before and one month after December 1, 2022) for a total of 50 patients. Data was collected via manual chart review. The primary endpoint was frequency of combination antibiotic therapy in each time period. Secondary endpoints included treatment success (defined as resolution of infection symptoms and lack of infection recurrence), in-hospital mortality, 30-day mortality, and 30-day infection recurrence. **Results:** Overall, baseline characteristics were similar between groups, the median age was 65 years, 64% of patients were male, 20% were immunocompromised based on prespecified criteria, the median Charlson comorbidity index was 5 (21% estimated 10-Year survival), and 76% of SM cultures were pulmonary isolates. Displayed in figure 1, combination therapy was given in 6 of 25 cases (24%) in the post-guidance group and zero patients in the pre-guidance group. Secondary endpoints of treatment success and all-cause mortality were similar between groups. Duration of therapy was similar between combination and non-combination therapy regimens (median 9 vs 10 days). Among patients who received combination therapy, all had ID consultation, 4 (66.7%) were admitted to the ICU, and 2 (33.3%) had treatment success. **Conclusions:** Patients treated for SM infection at our institution in the post-IDSA guidance period were more likely to receive combination therapy. A higher rate of treatment success was not observed in the post-IDSA guidance arm for SM infections. Limitations of this study include its small sample size and retrospective design, leading to inability to distinguish colonization from true infection. Additional studies are needed to evaluate the impact of combination antibiotic therapy on outcomes.

**Figure:**